# A Simplified Dynamic Strength Analysis of Cardboard Packaging Subjected to Transport Loads

**DOI:** 10.3390/ma16145131

**Published:** 2023-07-20

**Authors:** Damian Mrówczyński, Tomasz Gajewski, Tomasz Garbowski

**Affiliations:** 1 Doctoral School, Poznan University of Life Sciences, Wojska Polskiego 28, 60-637 Poznan, Poland; damian.mrowczynski@up.poznan.pl; 2 Institute of Structural Analysis, Poznan University of Technology, Piotrowo 5, 60-965 Poznan, Poland; tomasz.gajewski@put.poznan.pl; 3 Department of Biosystems Engineering, Poznan University of Life Sciences, Wojska Polskiego 50, 60-627 Poznan, Poland

**Keywords:** vertical random vibrations, compression strength, numerical modeling, finite element method, corrugated board packaging

## Abstract

The article presents a simplified method for determining the strength of corrugated board packaging subjected to dynamic transport loads. The proposed algorithm consists of several calculation steps: (1) a static analysis of the compressive strength of the package, (2) an analysis of random vibrations in the frequency domain used to determine the resonance frequencies and (3) a dynamic analysis of the package loaded with computed resonant frequencies. For this purpose, numerical models of the static compression test of the packaging before and after the dynamic analysis of the package subjected to general transport loads were developed. In order to validate the model, laboratory packaging compression tests were also performed for samples of boxes using three-layer cardboard. Due to this, it was possible to verify the numerical simulation results of the compression tests for several box geometries. This, in turn, allowed for the development of a method based on dynamic and post-dynamic (static) numerical analyses, permitting a high-accuracy determination of the resistance of the selected packaging to vibrations and dynamic loads. The results of the (experimentally validated) numerical analysis proved the usefulness of the simplified method presented herein for precise estimation of the load capacity of various packages dynamically loaded during transport.

## 1. Introduction

The requirements of the cardboard packaging are strongly related to the transport conditions to which a box will be exposed. Different packaging specifications are required for ocean transport in containers, local domestic transport and long-distance heavy truck transport. Depending on the model of transport, the safety factors can range from three to even eight [1]. Often, such high values are roughly assumed with a large overestimation, which is not supported by reliable research or an analysis of specific cases. Therefore, it is essential to optimize the weight of the packaging, especially now that the shipping industry is striving for sustainable development—zero waste, zero energy balance, etc. The first step for optimizing packaging should be understanding which factors play a crucial role in determining the strength of the packaging due to a particular load mode [2,3,4].

It is worth emphasizing that transport loads are, to a large extent, not static but dynamic loads; hence, high safety factors are used in such cases. In the corrugated packaging industry, the test known as the vertical random vibration (VRV) test is the basic tool that allows for assessing the resistance of a given package to dynamic loads [5]. It is particularly important to compare the load capacity of the packaging using a static box compression test (BCT) before and after the vertical random vibration test. The great advantage of this approach is the possibility to compare the results for different types of packaging. Conducted even for only one type of packaging, these tests allow for the elimination of packaging designs that would be defective in regard to dynamic loading.

However, for the companies that produce cardboard packaging, performing such tests in the factory requires special resources. This includes preparing many packaging samples with the necessary seasoning and performing the whole testing campaign using multiple series of tests, including BCTs of the packaging before the random vibration test, nominal VRV testing and BCTs of the packaging after the random vibration test.

Therefore, the use of modern numerical methods for the initial assessment of the suitability of the packaging design and for obtaining insight for the packaging prototype and/or cardboard grade selection seem to be very useful. The finite element method (FEM) is one of the most commonly used numerical methods for the mechanical analysis of structures [6,7]. The effectiveness of this method is confirmed by many scientific works and industrial implementations, including in the cardboard packaging industry [8,9,10,11,12,13,14,15,16]. The finite element method requires the use of detailed/accurate packaging geometry in order to obtain reliable calculation results. For instance, in [17] it was shown that the size/position of the opening and perforation patterns in packaging play crucial roles in determining the strength of the packaging. Moreover, reliable material data should be used. These data must reflect the mechanical properties of the cardboard from which the packaging is produced, and an adequate constitutive model must be adopted. Commercial products are available that enable the acquisition of the mechanical properties of cardboard and the implementation of the FEM calculations [18]. As shown in the literature, the commonly used empirical approaches for modeling the compression strength are not enough in complex cases [19].

It should be emphasized that a traditional dynamic analysis using the finite element method to model the VRV test in the time domain would be extremely computationally expensive. Therefore, in this paper, we proposed a method combined with a power spectral density (PSD) approach, which allowed us to solve a substitute dynamic problem in the frequency domain that identified a solution to the initial problem in the time domain. The PSD approach is a method that allows for simplification of the problem and is used effectively in many branches of mechanical engineering, including the automotive industry [20], aerospace [21], mechanics and machine construction [22], and biomedical engineering [23].

In the scientific literature, there are simplified methods for assessing the load capacity of packaging against static loads. An example of the work on the static load capacity of packaging can be found in the paper from Garbowski et al. [19], in which the McKee formula and its extensions were considered, and their effectiveness was compared with the numerical approach. Another example is an article on the effect of offsets [17] on the static load capacity of corrugated cardboard packaging. There are few studies that deal with the dynamic properties of packaging or corrugated boards [24,25]; hence, this work is of a particularly innovative/unique character.

Additionally, no papers on the use of the PSD method for estimating the strength in respect to the VRV test of the cardboard packaging have been found in the literature. Such examples are available in the field of mechanics and machine construction, for example in the article on the reliability of wind power turbines [26]. Therefore, in this work, a combination of a static and a dynamic test based on the PSD–VRV method was adopted to develop a fast and reliable algorithm for determining the strength of a corrugated box subjected to dynamic loads. To our knowledge, this has not been done before by other researchers, and therefore, this work offers a new contribution related to the effective estimation of the load capacity of cardboard packaging under dynamic loads.

## 2. Materials and Methods

### 2.1. Measurements of the Compressive Strength of Boxes

**Figure 1:**
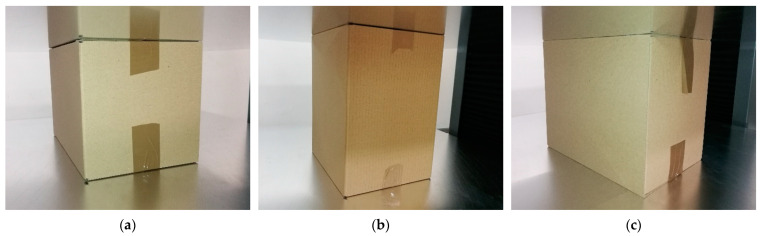
Selected samples of the considered packaging in the BCT machine just before the compression test. (**a**) box dimensions 250 × 250 × 150 mm; (**b**) box dimensions 300 × 200 × 450 mm; (**c**) box dimensions 300 × 200 × 420 mm.

In order to achieve reliable results from the numerical simulations, the results must be validated using the experimental data. Here, the numerical results received from the BCT were calibrated by comparing the outcome with the static compression tests of the boxes conducted in the laboratory on the press machine. The nominal press force was 10 kN with an accuracy of 0.1 N. The accuracy of applying the displacement was 0.001 mm. The cardboard used for the box samples was single-wall three-layered board with a 400 g/m^2^ grammage. The B flute was also used. The mean thickness of the cardboard was 2.85 mm. Three geometries of the FEFCO code F201 boxes were tested, namely, 250×250×150 mm, 300×200×250 mm and 300×200×450 mm. Five samples were prepared for each geometry of the box to obtain a proper statistical representation. The selected box samples inside the testing machine are presented in Figure 1.

Before the test, the packages were folded manually by one person, and the top and bottom flaps were taped since this is the standard practice and ensures box integrity (Figure 1). However, taping does not directly influence the BCT. The packages were conditioned according to the laboratory standard of TAPPI T402 [27], which determines the standard conditioning and testing atmospheres for paper, board, pulp hand sheets and related products. Therefore, a humidity of 50% and a temperature of 23 °C was set in the preconditioning chamber.

### 2.2. Measurements of the Vertical Random Vibration Test of Boxes

In order to determine and compare the performance of the packaging to the environmental vibration during transportation, the standard testing procedure was required. In this paper, the ISO 13355 standard was used [5]. The standard describes the method for testing the packaging in a vertical random vibration test. The method is more reliable and gives more realistic results than the sinusoidal vibration tests described in ISO 2247 [28] and ISO 8318 [29].

**Figure 2:**
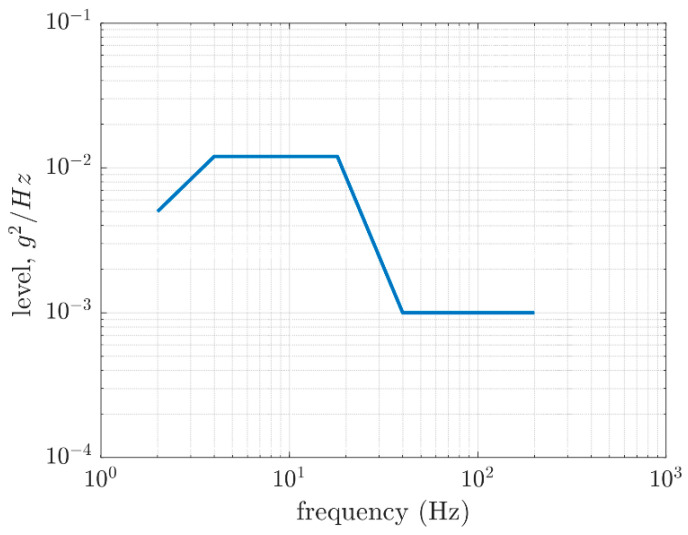
Power spectral density profile in the generic transportation from ISO 13355 [5] used in the paper.

In the ISO 13355 test, a vibrating table was excited for the effective frequency domain with a cardboard box sample placed on it. The setups of the test were predetermined, which applied, for example, to the duration of the test, the ambient temperature, the humidity and the acceleration of the power spectral density. During the test, the box sample cannot translate horizontally and must be closed. The vibration table must be stiff, and the gravity center of the specimen must be positioned in the center of the table. According to ISO 13355, if the experimental data for reproducing the effects of transportation are absent, the test duration and power spectral density of the vibration table may be assumed, as shown in Figure 2. Therefore, the data demonstrated in Figure 2 were used in the presented numerical study as the representative data for reproducing the environmental vibrations during transportation.

### 2.3. Numerical Modeling of the Resistance of the Cardboard Packaging Due to Transport Loadings

**Figure 3:**
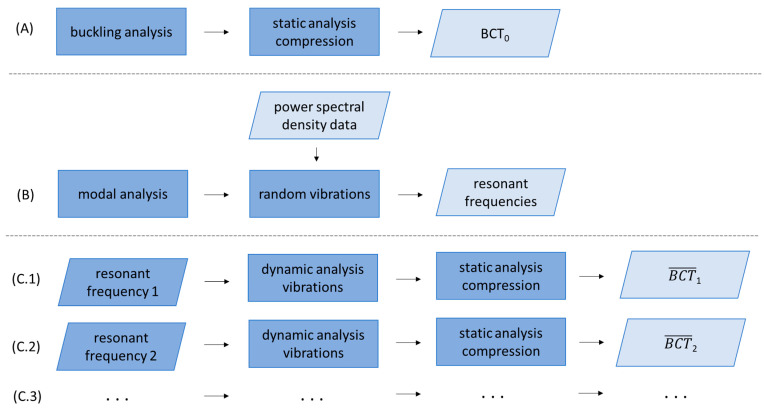
The scheme of the procedure for calculating the decrease in the load-bearing capacity of the packaging due to random vertical transport loading Steps from (**A**–**C.3**).

The overall aim of the paper is to derive a numerical tool for computing the changes in the compression strength of a particular box after representative environmental vibration during transportation. Therefore, as presented in Figure 3, the static finite element analysis of the stress-free state box was first performed and was preceded by a buckling analysis to determine the numerical imperfection field, in which was similar to [3,4]. From this step $BCT_{0}$ was determined, i.e., the compression strength of the box before the VRV test. Second, the modal analysis of the box was conducted and, together with the power spectral density (PSD) data, they were used as the input for the random vibrations analysis. The eigenmodes from the eigenfrequency (modal) analysis were used to compute the response variables, such as the stresses, strains or displacements. The numerical details about the method are available in [30,31,32]. In this step, the resonant frequencies, $\omega_{i}$ were determined.

Third, the displacement boundary condition with a particular ith resonant frequency, $\omega_{i}$, was applied to the bottom of the box in a dynamic analysis that simulated the vibrations using a fixed frequency. Next, after a selected number of cycles, the quasi-static compression test analysis was carried out numerically. From the last analysis, $\bar{BCT_{i}}$ was determined, i.e., the compression strength of the box after the VRV test in respect to ith resonant frequency, in which i depends on the number of the determined resonant frequencies from the random vibrations analysis.

**Figure 4:**
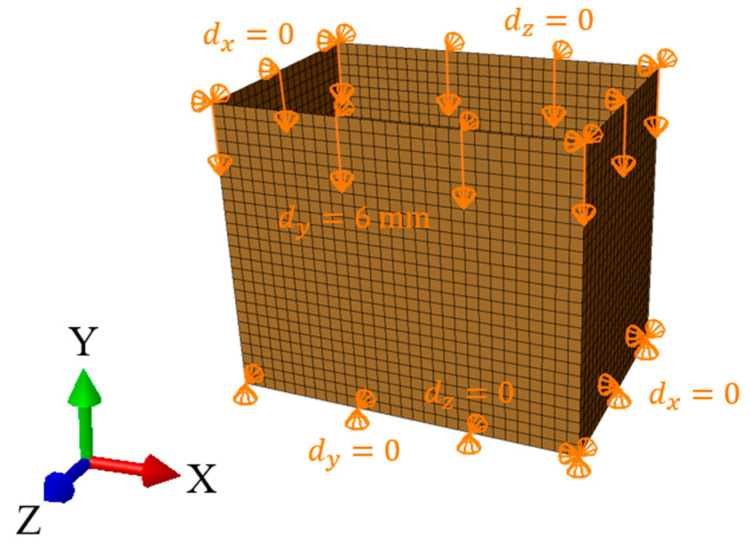
Finite element mesh and boundary conditions in the static analysis.

In the study, the static analysis of the box compression, as shown in Figure 3A, replicated the laboratory test conditions, which was similar to [3,16,17]. In the finite element method (FEM) analysis, only the load-bearing walls of the packaging were modeled (Figure 4). The bottom and top flaps were taken into account by applying appropriate boundary conditions. The out-of-plane displacements on the bottom and top edges of the sidewalls were blocked. The box compression test was simulated by applying a vertical displacement to the top edges of the box. The other details on the finite element mesh and constitutive modeling will be described at the end of this section.

The study of the vertical random vibrations in the time domain is a very dynamic process and easy to carry out in laboratory conditions. A vibration table and acceleration/displacement sensors are required to perform the test properly. On the contrary, the numerical counterpart of such a test modeled in the time domain is not trivial to simulate due to extremely high nonlinear boundary conditions, which changes with time. Thus, to compute the structural response of any model in the time domain, such boundary conditions could greatly decrease the numerical time step and make convergence difficult. Therefore, in this paper, the new approach for modeling the VRV test of corrugated board packaging was used, in which the modal analysis was used to determine the resonant frequencies for later use in the dynamic explicit finite element (FE) analysis with a stable vibration frequency, as shown in Figure 3B.

**Figure 5:**
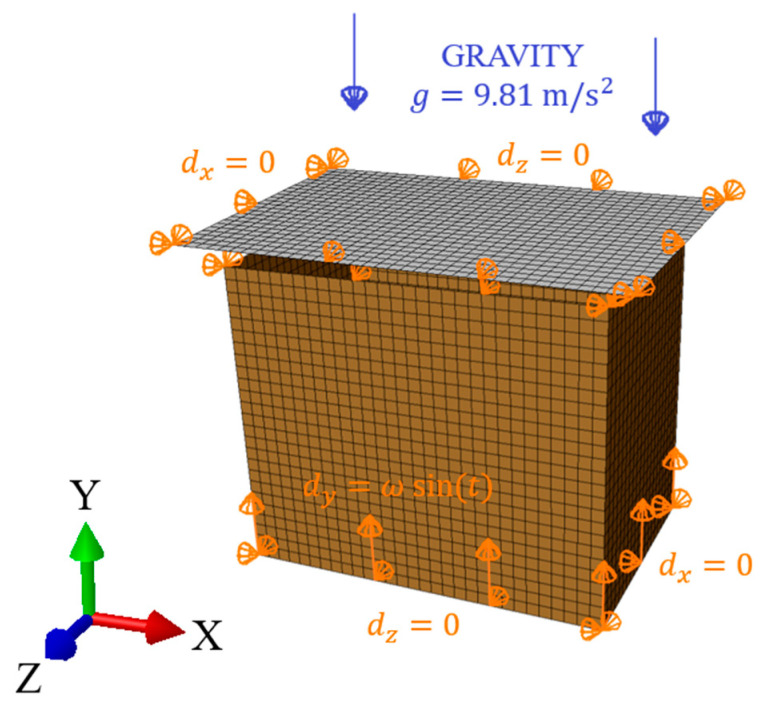
Finite element mesh and boundary conditions in the dynamic analysis.

Compared to the static model, a rigid plate was added to the dynamic explicit analysis to simulate the 8 kg load used in a real vertical random vibration test, as shown in Figure 3(steps C.1–C.3). The dynamic analysis consisted of two computational steps. In the first step, the model was loaded with gravity ($g=9.81\;m/s^{2}$), which caused the plate to fall onto the packaging. The packaging “held” the rigid plate by applying contact between these two modeled objects. As in the case of the static analysis, the out-of-plane displacements at the bottom and top edges of the packaging were blocked. The in-plane displacement of the plate was also blocked, as shown in Figure 5. In the second step, the bottom of the box vibrated in the time domain with a sinusoidal amplitude of 1 mm and a frequency obtained from the modal analysis.

The static, modal and dynamic analyses were carried out for one grade of a corrugated board. In each case, the assumed constitutive model was linear, elastic orthotropic material with a Hill plasticity [33]. The Hill model describes the behavior of the cardboard in the inelastic phase, which indirectly decreases the strength of the cardboard in machine direction. As shown in Table 1, the material constants used in the model to simulate the behavior of the 3B400 cardboard were presented. As shown in Table 1, the first columns contained elastic constants, where $E_{1}$ and $E_{2}$ are the modulus of elasticity, $\nu_{12}$ is Poisson’s ratio, $G_{12}$ is the in-plane shear stiffness, and $G_{13}$ and $G_{23}$ are the shear stiffnesses. In the last columns, the plastic constants of the material were presented, where $\sigma_{0}$ is the initial yield strength and $R_{11}$ is the yield strength factor in the machine direction of the corrugated board, according to the Hill potential [33].

The material data for the 3B400 cardboard were determined using the BSE system from FEMAT [33]. In the system, the properties were determined based on four mechanical tests in the cardboard machine and cross-machine directions. The corrugated board samples were prepared in the laboratory and conditioned in a climatic chamber, according to the TAPPI standard T402 [27]. At least ten samples of cardboard were used for each test to obtain the statistically representative material data.

In each case, four-node quadrilateral shell elements with full integration schemes were used to model the corrugated board box. This type of element was labelled as S4 according to Abaqus FEA [33]. Global mesh sizes of 8–10 mm were assumed, which resulted in a different number of degrees of freedom for each geometry case. In the 250×250×150 mm model, the number of elements was 2356 and the number of nodes was 2480. In the 300×200×250 mm model, the number of elements was 2500 and the number of nodes was 2600. In the 300×200×450 mm model, the number of elements was 4500 and the number of nodes was 4600. In the dynamic analyses, the rigid plate was modeled discreetly with 864 finite S4 elements and the rigid body properties were assigned to the elements.

## 3. Results

### 3.1. Static Compressive Strength of Boxes

In this section, the outcome of the laboratory compression tests and their numerical counterparts is shown in order to validate the numerical model of the corrugated board packaging. The validation of the mathematical modeling is an intrinsic part of computational mechanics and corroborates the obtained results.

**Figure 6:**
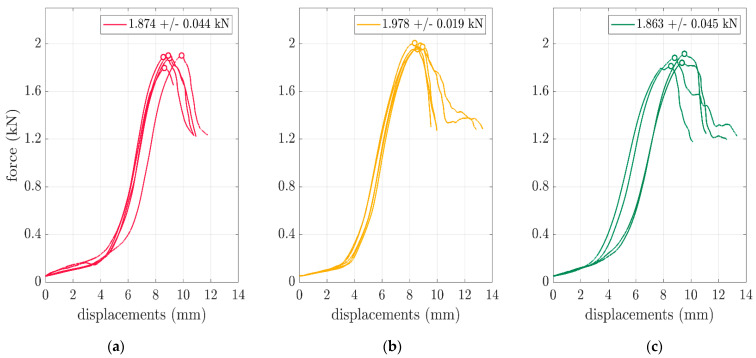
Box compression test results for the FEFCO F201 boxes: (**a**) 250×250×150 mm, (**b**) 300×200×250 mm and (**c**) 300×200×450 mm.

Therefore, as shown in Figure 6, the box compression test results were presented for the selected boxes of FEFCO F201 with various dimensions. The force vs. displacement curves were obtained for all the used box samples. The details of the testing procedure are described in Section 2.1. In the plots, the peak values were marked with circles. A relatively small spread of the maximal values can be observed. The individual and mean values with standard deviations are presented in the second and third columns of Table 2.

**Figure 7:**
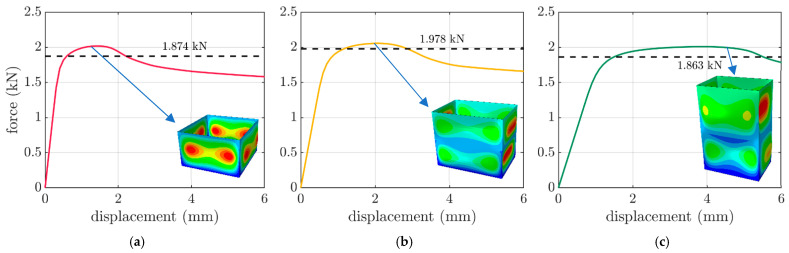
Box compression test results for the FEFCO F201 boxes from the numerical analyses: (**a**) 250×250×150 mm, (**b**) 300×200×250 mm and (**c**) 300×200×450 mm.

The counterpart cases were modeled using a static linear analysis by employing the finite element method. The details of the numerical models are described in Section 2.3. As shown in Figure 7, the numerically derived curves from the box compressions were presented for the selected boxes of FEFCO F201 with various dimensions. The dashed lines present the mean level of the obtained experimental values. As shown in the experimental plot (Figure 6), the first part was less inclined, and the real stiffness of the packaging is represented by the second part of the plot, i.e., the one that is more inclined. This two-phase plot effect was not observed in the computations (Figure 7) due to the ideal character of the packaging geometry. Additionally, the displacement field of the packaging at the maximal force are shown in Figure 7. The box compression strengths from the displacement control computations are presented in the last column of Table 2.

### 3.2. Vertical Random Vibrations of Boxes

The workflow of the study and the dynamic analysis concepts are presented in Section 2.3. Following the static computations, random response analyses were conducted for three dimensions of the boxes, i.e., 250×250×150 mm, 300×200×250 mm and 300×200×450 mm. Please note that for the 250×250×150 mm case, a wider frequency range was considered since the resonant frequency fell on the boundary of the interval at approx. 200 Hz. The assumed default power spectral density is presented in Figure 2.

**Figure 8:**
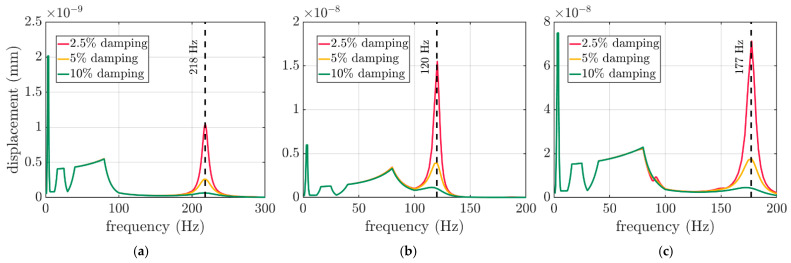
The outcome of the random response analyses for three damping levels with marked resonant frequency for boxes: (**a**) 250×250×150 mm, (**b**) 300×200×250 mm and (**c**) 300×200×450 mm.

In the random response analyses, three levels of damping were considered, i.e., 2.5%, 5% and 10% The computational outcome due to the modal analysis is demonstrated in Figure 8. The change in the damping factor determines the activity of a particular frequency, which is identified as the resonant frequency, $\omega_{i}$. Therefore, for each case of the boxes, three curves are presented in each subplot in Figure 8. The values of 218 Hz, 120 Hz and 177 Hz were identified as the resonant frequencies for the 250×250×150 mm, 300×200×250 mm and 300×200×450 mm boxes, respectively, as shown by the dashed vertical lines.

**Figure 9:**
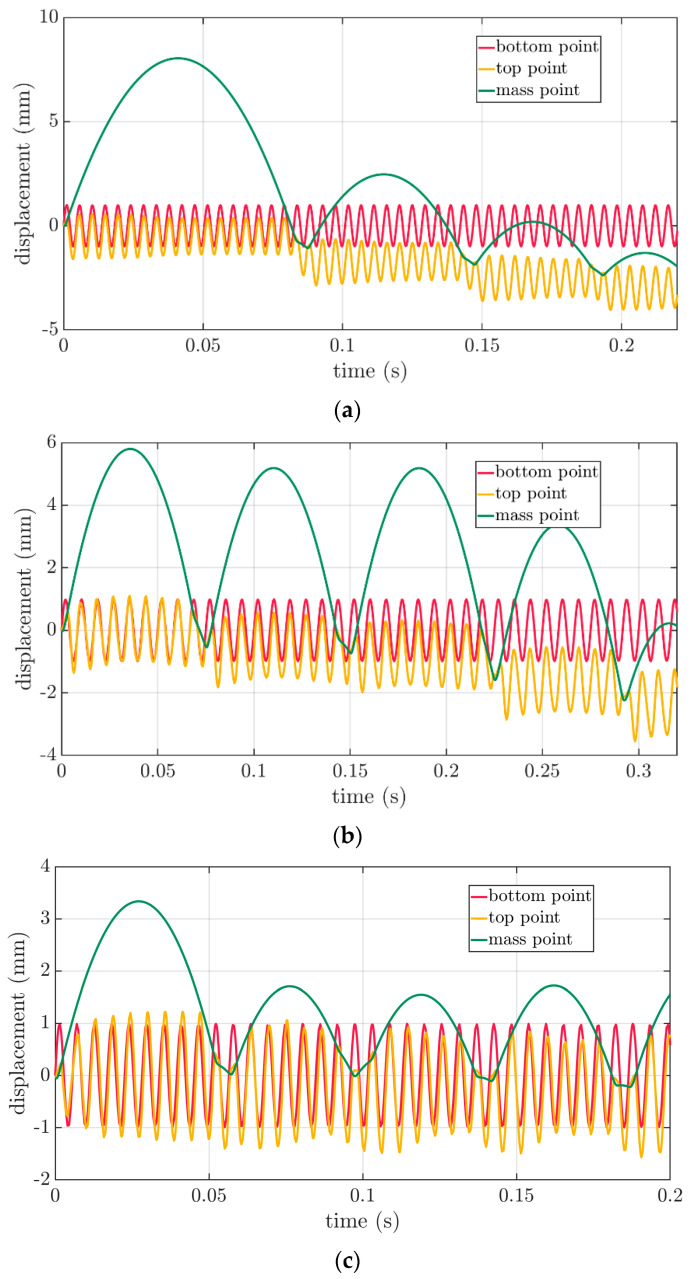
Vertical displacements of the bottom and top of the packaging and the plate for the FEFCO F201 boxes: (**a**) 250×250×150 mm, (**b**) 300×200×250 mm and (**c**) 300×200×450 mm.

For each box, the resonant frequencies were used to apply the time-dependent boundary conditions of the bottom of the box during the dynamic explicit FE analysis, as described in Section 2.3. As shown in Figure 9, the obtained results are shown in the time domain. Three types of curves were presented to represent the deformations of the packaging during the VRV test, i.e., the displacements of the bottom point of the packaging—red curve, the displacements of the top point of the packaging—yellow curve and displacements of the mass point of the rigid plate—green curve.

### 3.3. The Change of the Load Capacity of the Box as a Result of the Random Vibration Test

In the simplified method presented in the article, the resonant frequencies $\omega_{i}$ were determined, which excited the vibrations of the bottom of the packaging with a regular sine amplitude. The vibrations accelerated the rigid plate at the top of the packaging and elevated it until the plate separated the packaging. The plate fell and bounced off the packaging repeatedly. After each impact, the compression strength of the box was verified computationally using a static FE analysis and taking into account the non-zero state of stress. Due to the accumulation of damage caused by the plastic deformation inside the packaging, the box compression test curves differed from the outcome of the stress-free FEM static analysis.

**Figure 10:**
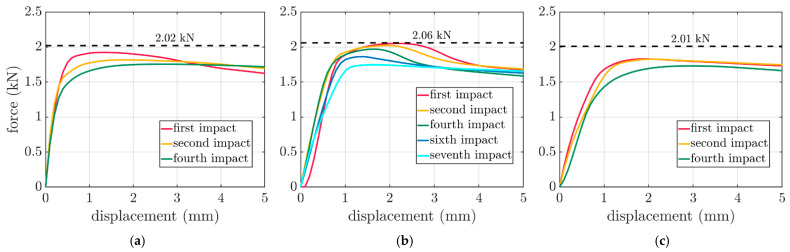
Box compression test results after a different number of impacts for: (**a**) 250×250×150 mm, (**b**) 300×200×250 mm and (**c**) 300×200×450 mm boxes.

The results of the FEM computations after a different number of impacts for the boxes considered in the paper are presented in Figure 10 for the 250×250×150 mm, 300×200×250 mm and 300×200×450 mm boxes. In each subplot, the dashed curve demonstrates the compression strength level from the stress-free FEM static analysis, while the colors represent the selected force–displacement curves after a particular number of impacts.

**Figure 11:**
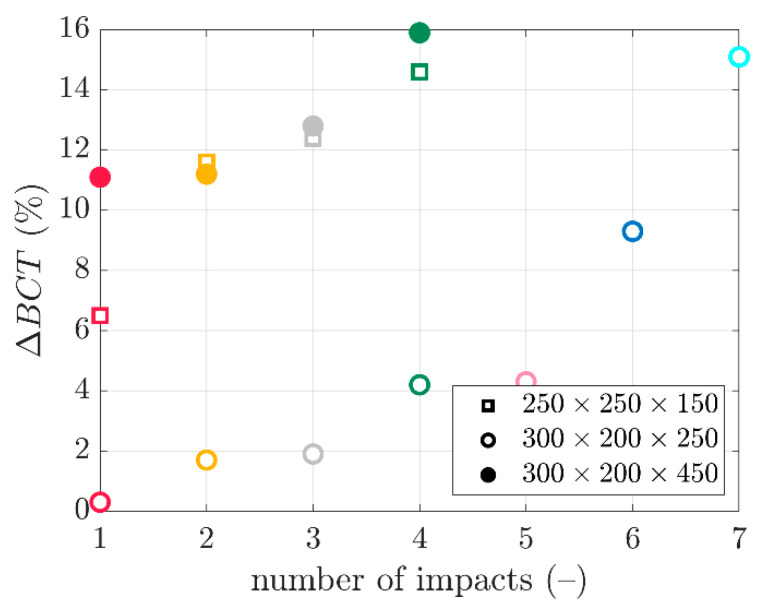
The decrease in the load-bearing capacity due to random vertical transport loading for different box dimensions and the number of impacts using a rigid plate.

The data from Figure 10 were summarized and are confronted in Figure 11. The peak values from Figure 10, i.e., the box compression strengths after the dynamic analysis of the packaging subjected to transport loadings, were divided by the counterpart static strengths before the VRV test simulation. Therefore, the ΔBCT was computed for a particular box and a different number of impacts. Thus, the vertical axis represents the ΔBCT, while the horizontal axis expresses the number of impacts of the rigid plate with the packaging. The empty square markers demonstrate the results for the 250×250×150 mm box. The filled circle markers summarize the results the for 300×200×250 mm box. The empty circle markers present the results for the 300×200×450 mm box.

## 4. Discussion

The validation of the numerical calculations in comparison with the results of the performed test reinforced the credibility of the computation results. Therefore, the validation was conducted on the compression tests from the considered packaging. As shown in Figure 6, the tests performed on the hydraulic press showed a very low discrepancy, i.e., not less than 0.05 kN in relation to the values of 1.874 kN, 1.978 kN and 1.863 kN for the 250×250×150 mm, 300×200×250 mm and 300×200×450 mm boxes, respectively. Moreover, as shown in Figure 7, based on these results, the performed validation of the numerical models showed a relatively low error. The errors of 6.6%, 4.0% and 7.9% were obtained and are graphically presented in Figure 7. Based on the above errors, it can be concluded that the computational approach was verified. A successful validation corroborated the rest of the results of the numerical calculation to a greater extent.

To compute the VRV response, the resonant frequencies ω were computed. As shown in Figure 8, the change in the damping factor determined the activity of a particular frequency, which was identified as the resonant frequency. The results in the graphs, as shown in Figure 8a–c, were consistently similar to each other (the same FEFCO code box). However, the change in the dimensions of the packaging determined the position of the resonant frequency for a particular case.

One of the most interesting results was presented in Figure 9, which demonstrated the interactions between the reflecting rigid plate and the induced vibrations in the packaging. As shown in Figure 9a, the lowest box interaction was presented, i.e., the 250×250×150 mm case. In this case, the rigid plate was initially raised relatively high at about 8 mm. The first (0.08 s) and subsequent impacts (0.15 s and 0.19 s) significantly deformed the packaging since the top point of the plot (yellow curve) successively decreased its level after each bounce, which was similar to the successive positions of the rigid plate (green curve).

The medium height box interaction was presented in Figure 9b, i.e., the 300×200×250 mm case. In this case, the plate was initially raised to a height of 6 mm. Here, after the first impact (0.075 s), the deformation was not so significant. Similar to the next bounce (0.15 s), a slight lowering of the upper edge of the packaging (yellow plot) was noticed. Significant increases in packaging deformation were only noticed after the third (0.225 s) and fourth reflections (0.29 s).

The highest box interaction was presented in Figure 9c, i.e., the 300×200×450 mm case. In this case, the first elevation of the plate was relatively low at about 3.3 mm. Each reflection was similar to the other and much lower than the first plate elevation at about 1.6–1.8 mm. In this case, the deformation of the upper part of the packaging edge (yellow curve) was not so visible for the first four reflections (0.06 s, 0.095 s, 0.14 s and 0.18 s).

The compressive strength curves of the boxes due to transport loading after each impact are summarized in Figure 10. The characters of the curves compared for increasing the number of impacts were different, and they did not scale linearly. Additionally, there were no similarities between the boxes, as shown in the comparison between Figure 10a,c. Both presented strength curves after the first, second and fourth impacts. It can be observed that for the yellow and green curves, the changes in the shape were completely different.

The synthetic data from Figure 10 are shown in Figure 11, in which the trends in the compressive strength decrease due to transport loading can be observed for a different number of impacts. Figure 11 demonstrates that the medium height packaging, the 300×200×250 mm box, was the most resistant to the BCT reduction due to the transport load. That is, the ΔBCTs were the lowest for these cases (empty circles). From the first to the fifth impact, the ΔBCT did not exceed 5%. Only after the sixth and seventh impacts did the ΔBCTs equal nearly 10% and 15%, respectively. Moreover, the lowest packaging, the 250×250×150 mm box, demonstrated a relationship close to the linear decrease in the BCT with an increasing number of impacts. Here, the ΔBCT values ranged from approx. 6% to 14% for the first to fourth impacts (empty squares). Moreover, for the highest packaging, i.e., the 300×200×450 mm box, the obtained values were similar to those for the lowest packaging, although this packaging showed the highest ΔBCT values. For example, for the first impact obtained a value of 11% (filled circles).

The summary plot in Figure 11 compares the resistance of the packaging to the transport loads by comparing the point graphs created due to the simplified computational method presented in the article. As shown earlier, a few analyses of the impacts of the plate against the packaging, according to the presented method, were enough to determine which packaging had a better resistance to the equivalent transport loads.

## 5. Conclusions

In this paper, a simplified method for calculating the resistance of corrugated cardboard packaging to vertical vibrations during transport was presented. Three geometries of FEFCO F201 packaging, composed of the same type of corrugated cardboard, were modeled. Dynamic analyses were carried out to check their resistance to vertical vibrations. The created models were validated using the experimental results from the static compression. In the first stage of the computations, a buckling analysis was performed, and then the static load capacity was determined. In the second stage of the simulation, a modal analysis was performed, which determined the resonant frequency of the boxes. Then, a rigid plate was modeled, which was loaded with a gravity force and set in motion by vibrating of the bottom edges of the packaging using a resonant frequency. After each impact between the plate and the packaging, the compressive strength of the box was measured, which allowed us to calculate the decrease in the load capacity due to the dynamic load.

Even though the analyzed boxes represented the same type of box, the paper shows that each corrugated cardboard packaging reacted differently to vertical vibrations during transport. As expected, each successive impact of the rigid plate reduced the compression strength of the box, but depending on the case, the reduction varied. The BCT value in each case decreased by several percentage values. Due to the novel method proposed, the alternative packaging designs can be verified in regard to its resistance to generic transport loading using a simplified numerical approach. Moreover, the main novelty of the numerical method is that it does not require the time domain to be taken into consideration to identify the resonant frequency. However, with the proposed surrogate problem, the resonant frequencies may be identified in the frequency domain, making it more efficient and easier to be solved numerically.

## Figures and Tables

**Table 1:** Material constants used in the constitutive model of the corrugated board 3B400.

$E_{1}$	$E_{2}$	$\nu_{12}$	$G_{12}$	$G_{13}$	$G_{23}$	$\sigma_{0}$	$R_{11}$
(MPa)	(MPa)	(–)	(MPa)	(MPa)	(MPa)	(MPa)	(–)
1545.1	843.7	0.402	312.2	7.1	23.1	2.0	0.951

**Table 2:** Box compression test results for the FEFCO F201 boxes with their numerical counterparts.

	Tests		Computations
Dimensions	${BCT}_{0}$ (kN)	$\mathrm{Mean}\;{BCT}_{0}$ with Standard Deviation (kN ± kN)	${BCT}_{0}$ (Error to Exp.) (kN)
250×250×150	1.797	1.874 ± 0.044	2.02 (6.6%)
	1.902		
	1.888		
	1.879		
	1.902		
300×200×250	1.956	1.978 ± 0.019	2.06 (4.0%)
	2.006		
	1.970		
	1.984		
	1.974		
300×200×450	1.840	1.863 ± 0.045	2.01 (7.9%)
	1.882		
	1.813		
	1.915		

## Data Availability

The data presented in this study are available on request from the corresponding author.
